# Identifying barriers and utility of obtaining ambulatory blood pressure monitoring in a pediatric chronic kidney disease population

**DOI:** 10.1186/s12887-023-04304-7

**Published:** 2023-09-16

**Authors:** Sanober Sadiq, Elizabeth Black, Aris Oates

**Affiliations:** 1 Department of Pediatrics, University of California, San Francisco, 550 16th Street, 4th Floor, 3214, San Francisco, CA 94143 USA; 2 Department of Pediatrics, University of California, San Francisco – Fresno, Fresno, CA USA

**Keywords:** Chronic kidney disease, Hypertension, Ambulatory blood pressure monitoring, Masked hypertension, White coat hypertension

## Abstract

**Background:**

Hypertension is a prevalent complication of Chronic Kidney Disease (CKD) and Ambulatory Blood Pressure Monitoring (ABPM) is the gold standard for diagnosis. The aim of our study was to assess the usefulness of obtaining ABPM and to identify barriers to ABPM in this pediatric patient population.

**Method:**

In this retrospective analysis of patients with CKD stage 3–5 who were seen in one academic medical center’s outpatient Pediatric Nephrology clinics between 2018 and 2021, we performed logistic regression to evaluate for associations between demographic factors and odds of having an ABPM.

**Result:**

Among 96 patients included in the study, 48 patients carried a diagnosis of hypertension. 31 patients had ABPM performed with usable data. In those who had ABPM done, 21 had normotension and 10 had undertreated hypertension. Our study also showed 1 had masked hypertension and 5 had white coat hypertension or effect. We did not find a statistically significant difference in those who did or did not undergo ABPM evaluation based on gender, previous diagnosis of hypertension, distance from clinic, language preference, or racial or ethnic identity.

**Conclusion:**

ABPM is a useful tool in our CKD population for the diagnosis and management of hypertension. We did not identify specific barriers to ABPM in our CKD population, and there were no differences in patients who obtained ABPM when looking at specific demographic and disease characteristics. Given these findings, we recommend focusing on areas of future improvement in spheres of patient and provider education as well as better quantification using surveys to further illuminate barriers.

## Introduction

Approximately 1% of all patients with chronic kidney disease (CKD) are children and adolescents, with CKD affecting 15-74.5 children per million globally [1]. Hypertension is found in a considerable proportion of pediatric patients with CKD and End Stage Renal Disease (ESRD), ranging from 50 to 80% depending on the stage of disease [1, 2], with a prevalence 10-fold higher than in the general pediatric population. The Chronic Kidney Disease in Children (CKiD) study is a North American, multi-center, prospective study of CKD in children which found 54% of patients with CKD had elevated blood pressure (BP) and/or a history of hypertension on anti-hypertensives. [3]. Hypertension in CKD is primarily due to activation of the renin–angiotensin system which results in vasoconstriction, sodium and water retention, and activation of the sympathetic system. Chronically elevated BP causes additional renal damage through micro vascular changes and further progression of CKD. Hence, there is a higher prevalence of hypertension during the later stages of CKD [1, 4] as high as 80% in CKD stages 3–5 [5]. It is especially important to control hypertension in this population as it can lead to an even further accelerated decline in GFR [3] and is an independent risk factor for progression of CKD [6].

Repeated studies have shown the superiority of ambulatory blood pressure monitoring (ABPM) evaluations for BP assessment. Office BP measurement alone can misclassify BP status in over 40% of subjects, when compared to ABPM evaluation [7]. Home BP monitoring, though commonly done in pediatric CKD populations, does not have sufficient sensitivity or specificity to be used as a single diagnostic test [8, 9]. ABPM evaluations have superiority over clinic BP measurements to diagnose masked hypertension (controlled BP in an office setting with elevated BP readings outside of the office) [10]. By intensifying BP control as assessed using ABPM, there is a substantial benefit with respect to kidney function seen among children with CKD [2, 11]. Thus, the American Academy of Pediatrics and Kidney Disease Outcome and Quality Initiative guidelines both recommend 24-hour ABPM at least annually for the detection of white coat (elevated BP in office setting with normal BP reading outside of the office) and masked hypertension in all children with CKD and to start treatment for hypertension [12–14]. The updated 2022 statement from the American Heart Association further highlights this importance and provides new data on ABPM results linking with target organ damage [15].

Despite the evidence and recommendations, ABPM is not routinely obtained in the CKD population, and there have been studies done to identify barriers to obtaining ABPM. A focus-based group study on English and Spanish preferred language adults showed patients being concerned about cost of the procedure and discomfort while wearing ABPM device [16]. Another international study similarly showed discomfort and cost to be the most common barriers [17]. A physician-based study showed concern about cost, lack of resources, and lack of proper education to be common reasons for not ordering ABPM [18]. These studies were done in non-CKD, adult populations. Studies on barriers to ABPM in both CKD and pediatric populations are limited.

The aim of our study is to identify any additional barriers to ABPM in pediatric patients with CKD and to assess the utility of the study in this population.

## Materials and methods

### Study population

Retrospective chart reviews were done on all patients with CKD stage 3–5 who were seen in the Pediatric Nephrology ambulatory clinics of one academic medical center between January 2018 and December 2021. Inclusion Criteria were patients with CKD stage 3–5, age 5 or greater, and height > 120 cm. Patients on dialysis or with a history of kidney transplant were excluded. Approval was obtained from the Institutional Review Board.

### Office and ambulatory blood pressure monitoring

Office blood pressures were measured using an oscillometric device and 1–2 readings were obtained.

Patients were referred by their primary nephrologists to the ABPM clinic at our center. The ABPM visit was conducted either in person or by telehealth. Office blood pressure readings were collected from the ABPM visit if in person or from the most recent nephrology visit.

ABPM was performed using a Spacelab 90217 monitor. The ABPM was either placed on the non-dominant arm using an appropriately sized cuff by the pediatric nephrology nurse practitioner or by a family member with provider instruction if placement was done through telehealth. For patients who were seen by telehealth, the ABPM was mailed to their home with an included return label. BP was measured every 20 min during the day and every 30 min at night during a 24-hour period. Once ABPM was done, it was returned by mail. Usable data was defined as having at least 40 to 50 readings or 70% successful readings for a full 24-hour period.

### Definition of clinical parameters

We used normative data for ambulatory BP as presented by Wuhl et al. [19]. Using both oscillometric clinical and 24-hour readings, ambulatory BP status was categorized based on the 2022 American Heart Association Guidelines definitions with age < 13 years and ≥ 13 years used as cutoffs [15]. For less than 13 years of age, Normal BP was defined as casual BP < 95th percentile with mean Ambulatory BP < 95th percentile. Ambulatory hypertension was defined as casual BP ≥ 95th percentile with mean ambulatory BP ≥ 95th percentile. White Coat Hypertension was defined as casual BP ≥ 95th percentile with mean ambulatory BP < 95th percentile. Masked Hypertension was defined as casual BP < 95th percentile with mean ambulatory BP ≥ 95th percentile. For ≥ 13 years of age, normal BP and White Coat Hypertension were defined as mean Ambulatory Blood Pressure < 125/75 mmHg over 24 h period and Masked Hypertension and Ambulatory Hypertension were defined as mean Ambulatory Blood Pressure > 125/75 over 24 h period. Patients who were on antihypertensive medication and met criteria for white coat hypertension were considered to demonstrate a white coat effect.

Patients with a previous history of hypertension were considered to have well-controlled hypertension based on casual BP < 95th percentile and mean ambulatory BP < 95th percentile. Conversely, patients were considered to have under-treated hypertension if they had mean ambulatory BP ≥ 95th percentile or > 125/75.

### Statistics

We examined the association between access to ABPM and demographic characteristics including age, gender, preferred language (English, Spanish or other), race/ethnicity, physical distance from the clinic, presence of social concerns as documented by licensed clinical social worker, number of visits per year, glomerular versus non-glomerular etiology of CKD, CKD stage, and existing diagnosis of hypertension using logistic regression models.

Stata 14 (StataCorp, TX: LLC) was used for the performance of all statistical analyses. P-values < 0.05 were considered statistically significant for all analyses.

## Results

Ninety-six patients were included in this study; 54 were males and 42 were females. Forty-eight (50%) had a prior diagnosis of hypertension. ABPM was performed in 33 patients out of which 31 patients had ABPM with usable data (32%). Twenty out of those 33 patients had a diagnosis of hypertension before ABPM was performed, and 18 of these contributed usable data. Of the patients with an ABPM performed with prior diagnosis of hypertension, 9 had confirmed undertreated hypertension (50%), 5 had well-controlled hypertension (28%), 3 were diagnosed with white coat effect (16%) and 1 had masked hypertension (6%). (Table 1)

**Table 1:** ABPM results

No Previous Hypertension Diagnosis (n = 13)	N (%)
Normal	11 (85%)
Masked Hypertension	0 (0%)
White Coat Hypertension	2 (15%)
Confirmed Suspected Hypertension	0 (0%)
Prior Hypertension Diagnosis (n = 18)	N (%)
Well Controlled Hypertension	5 (28%)
Masked Hypertension	1 (6%)
White Coat Effect	3 (16%)
Confirmed Undertreated Hypertension	9 (50%)

Results for those undergoing ABPM without a prior history of hypertension are also shown in Table 1.

The possible demographic barriers that we analyzed were gender, race/ethnicity, language, clinic distance, number of visits, CKD stage, CKD etiology, and documented social concerns. None of the demographic features that we evaluated were statistically associated with access to ABPM (Table 2).

**Table 2:** Odds of ABPM by demographic characteristics

Characteristics	Number (%)	ABPM (%)	Odds Ratio	P-Value	95% CI
**Gender**					
Male Female	54 (56%) 42 (44%)	22 (40%) 11 (26%)	Reference 0.52	Reference 0.14	Reference 0.21–1.23
**Age (years)**	Mean: 16 IQR: 13–19.5	Mean: 17.2 IQR: 15–21	1.07	0.19	0.97–1.18
**Race/Ethnicity**					
White Latinx Asian Black More than one Unknown	23 (24%) 45 (47%) 9 (9%) 9 (9%) 3 (3%) 7 (7%)	9 14 3 1 1 5	Reference 0.70 0.77 0.19 0.77 3.89	Reference 0.51 0.76 0.15 0.85 0.30	Reference 0.25–2.00 0.15–3.93 0.02–1.83 0.06–9.89 0.62–24.52
**Language**					
English Spanish Other	70 (73%) 24 (25%) 2 (2%)	24 9 0	Reference 1.15 -	Reference 0.78 -	Reference 0.44–3.01 -
**Clinic Distance**					
≤ 1 h > 1 h	66 (69%) 30 (31%)	23 10	Reference 0.93	Reference 0.885	Reference 0.38–2.33
**Number of Visits**					
1–2 3–4 5–6 >7	36 (38%) 44 (46%) 11 (11.6%) 4 (4.3%)	9 19 4 1	Reference 2.28 1.71 1	Reference 0.09 0.46 1.0	Reference 0.87–5.96 0.41–7.25 0.09–19.87
**CKD Stage**					
CKD 3 CKD 4 CKD 5	52 (54%) 22 (23%) 22 (23%)	18 7 8	Reference 0.88 1.08	Reference 0.82 0.89	Reference 0.30–2.56 0.38–3.05
**CKD Etiology**					
Glomerular Non- Glomerular	27 (29%) 66 (70%)	8 24	Reference 1.36	Reference 0.54	Reference 0.52–3.57
**Social Concerns**					
None noted Concerns noted	56 (65%) 30 (35%)	22 10	Reference 0.77	Reference 0.59	Reference 0.31–1.96

## Discussion

ABPM was not routinely done in our CKD population despite 50% having a known diagnosis of hypertension. Of those who did have an ABPM, 1/31 or 3% were diagnosed with masked hypertension which is consistent with other published data on the importance of annual ABPM screening. Since these ABPMs were performed prior to the updated 2022 definitions [15], load was used in the clinical interpretation by the provider. Based on prior definitions of ambulatory hypertension, 11 patients required intervention. Consistent with the CKiD study [20, 21] a significant percentage of our population required intervention including increasing their existing anti-hypertensive medication(s) or starting a new anti-hypertensive medication. There were also 5 patients who were diagnosed with white coat hypertension or effect and therefore were able to avoid increased medication burden and potential side effects. These numbers reinforce and further support the utility of obtaining an ABPM in the CKD pediatric population. Our gap in ABPMs performed despite the guidelines, matches experience of patients included in CKiD study [22].

Our study is one of the first to investigate specific ABPM barriers beyond ethnic and racial identity in the pediatric CKD population. We selected barriers identified by other adult studies as well as other cited barriers in access to care. Variables such as distance from clinic and number of visits were not identified to be statistically significant barriers to obtaining ABPM. We also investigated social concerns (e.g., transportation insecurity, anxiety, depression) as being a barrier to obtaining ABPM with no association noted. In addition, our study did not identify ethnic and racial identity as being a barrier to ABPM use, in contrast to a recent study by Pagi et al. who found ABPM was performed less frequently in Black children versus White and Hispanic children [23]. Our study did not identify cost to be a reason as the overwhelming majority of patients had insurance that would cover the cost of ABPM.

In evaluating the specific barriers to pediatric ABPM, while we did not find specific barriers to ABPM, we have helped focus work in other areas that can help improve the gap in this important tool. There is a need for further studies to be done in the pediatric CKD populations to identify barriers to ABPM to help increase their usage. Additional exploration into patient transportation and financial insecurity, provider-based ordering and recommendation factors, and patient education and perspectives may reveal additional barriers that have not yet been described.

The strength of this study is that it is one of few studies examining the prevalence of and potential barriers to ABPM in the pediatric CKD population [20, 21, 23]. Many adult studies have described the benefits of obtaining ABPM in an adult population [24, 25], very few pediatric studies exist that examine both the benefits of and the barriers to ABPM utilization in a pediatric CKD population in real life [23]. This is also one of the few retrospective studies including only a pediatric CKD population. Our study also looked at the prevalence of masked hypertension and white coat hypertension in this population cohort and even though it was a retrospective study, it did provide a snapshot on utilization of ABPM in CKD population. Our study was limited in terms of small sample size when compared to other pediatric studies [23] and in investigating long term effects given the design of the study focused on chart review from a four-year time span. The prevalence of masked hypertension in our study was low for CKD which might be due to small number of children getting ABPM and criteria used to define masked hypertension compared to other studies [20]. It also did not include pediatric patients whose height was less than 120 cm given limitations in equipment and lack of normative data, though a prior pediatric study was able to perform ABPM in infants and toddlers [26]. Additionally, future analysis could be undertaken to assess ABPM control as defined by 24-hour Mean Arterial Pressure to < 50%th percentile [14, 15]. CKD stages 1–2 were not included and exploring this population further could potentially prove informative. We also did not have a patient or provider-based survey and therefore were not able to identify subjective barriers to ABPM. Previous studies have noted discomfort while wearing ABPM to be one of the most common patient-associated barriers [16, 17]. Only 2 of the patients in our study had unusable data. A detailed social evaluation in this population cohort could potentially yield additional understandings of patient associated barriers.

In our study, we found ABPM was a beneficial tool in diagnosing masked hypertension and white coat hypertension and therefore helpful in preventing CKD progression and extra medication burden. It also showed that approximately 50% of patients with prior diagnosis of hypertension were inadequately controlled. We did not identify any barriers to ABPM use, and we recommend focusing on areas of future improvement in spheres of patient and provider education as well as better quantification using surveys to further illuminate barriers and increase rates of ABPM monitoring. ABPM is more cost-effective than dialysis and renal transplantation and therefore should be performed routinely in CKD population to delay CKD progression and improve health outcomes.

## Data Availability

The datasets used and analyzed during the current study available from the corresponding author on reasonable request.
